# Hybrid Neuroprosthesis for the Upper Limb: Combining Brain-Controlled Neuromuscular Stimulation with a Multi-Joint Arm Exoskeleton

**DOI:** 10.3389/fnins.2016.00367

**Published:** 2016-08-09

**Authors:** Florian Grimm, Armin Walter, Martin Spüler, Georgios Naros, Wolfgang Rosenstiel, Alireza Gharabaghi

**Affiliations:** ^1^Division of Functional and Restorative Neurosurgery, Centre for Integrative Neuroscience, Eberhard Karls University TuebingenTuebingen, Germany; ^2^Department of Computer Engineering, Wilhelm Schickard Institute for Computer Science, Eberhard Karls University TuebingenTuebingen, Germany

**Keywords:** functional electrical stimulation, robot-assisted rehabilitation, brain-robot interface, brain-machine interface, brain-computer interface, functional restoration, motor recovery, upper-limb assistance

## Abstract

Brain-machine interface-controlled (BMI) neurofeedback training aims to modulate cortical physiology and is applied during neurorehabilitation to increase the responsiveness of the brain to subsequent physiotherapy. In a parallel line of research, robotic exoskeletons are used in goal-oriented rehabilitation exercises for patients with severe motor impairment to extend their range of motion (ROM) and the intensity of training. Furthermore, neuromuscular electrical stimulation (NMES) is applied in neurologically impaired patients to restore muscle strength by closing the sensorimotor loop. In this proof-of-principle study, we explored an integrated approach for providing assistance as needed to amplify the task-related ROM and the movement-related brain modulation during rehabilitation exercises of severely impaired patients. For this purpose, we combined these three approaches (BMI, NMES, and exoskeleton) in an integrated neuroprosthesis and studied the feasibility of this device in seven severely affected chronic stroke patients who performed wrist flexion and extension exercises while receiving feedback via a virtual environment. They were assisted by a gravity-compensating, seven degree-of-freedom exoskeleton which was attached to the paretic arm. NMES was applied to the wrist extensor and flexor muscles during the exercises and was controlled by a hybrid BMI based on both sensorimotor cortical desynchronization (ERD) and electromyography (EMG) activity. The stimulation intensity was individualized for each targeted muscle and remained subthreshold, i.e., induced no overt support. The hybrid BMI controlled the stimulation significantly better than the offline analyzed ERD (*p* = 0.028) or EMG (*p* = 0.021) modality alone. Neuromuscular stimulation could be well integrated into the exoskeleton-based training and amplified both the task-related ROM (*p* = 0.009) and the movement-related brain modulation (*p* = 0.019). Combining a hybrid BMI with neuromuscular stimulation and antigravity assistance augments upper limb function and brain activity during rehabilitation exercises and may thus provide a novel restorative framework for severely affected stroke patients.

## Introduction

Standard of care leaves the majority of stroke survivors with a dysfunctional upper extremity and, consequently, with a long-term dependency on others for activities of daily living (Jørgensen et al., 1999; Dobkin, 2005; Feigin et al., 2008). Attempts to improve recovery in this patient group are numerous and embrace advanced rehabilitation technology for motor re-learning such as brain-interface based neurofeedback training (Ang et al., 2015; Morone et al., 2015; Pichiorri et al., 2015), robot-assisted rehabilitation devices (Lo et al., 2010; Klamroth-Marganska et al., 2014) and activity-dependent neuromuscular stimulation techniques (Thrasher et al., 2008; Oujamaa et al., 2009; Mann et al., 2011). Recent approaches combine these different methods in a bid to maximize the overall benefits (Meadmore et al., 2014; Brauchle et al., 2015; Hortal et al., 2015; Grimm and Gharabaghi, 2016). However, there is still a critical need in the rehabilitation community to provide options for stroke patients with chronic impairments. In this context, movement-related desynchronization (ERD) in the contralateral sensorimotor cortex has been shown to be compromised in stroke patients compared to healthy controls; notably, the more severe the patient's motor impairment, the less beta-band ERD (Rossiter et al., 2014). Accordingly, increasing this oscillatory modulation range again would provide a therapeutic target for a restorative training approach.

In the present proof-of-principle study, we explored an integrated approach for providing assistance as needed to amplify the task-related range of motion (ROM) and the movement-related brain modulation during rehabilitation exercises of severely impaired patients; we have, therefore, combined different rehabilitation tools: brain-controlled neurofeedback training, an upper limb multi-joint exoskeleton, and activity-dependent neuromuscular electrical stimulation (NMES). These different components served the following goals: The brain-controlled neurofeedback training based on motor imagery has recently been shown to increase task-related oscillatory modulation, specifically in the beta-frequency band, in correlation with corticospinal excitability (Kraus et al., 2016a) and motor learning (Naros et al., 2016a). Moreover, previous findings indicated that NMES amplifies both cortical ERD (Müller et al., 2003) and excitability when combined with motor imagery (Reynolds et al., 2015) or volitional effort (Stein et al., 2013). More specifically, during NMES movement a prominent ERD was found similar to that observed after active or passive movements suggesting that the sensorimotor processing during NMES involves some of the processes which are also involved in voluntary hand movements (Müller et al., 2003). Finally, multi-joint gravity compensation of the upper extremity has recently been shown to increase the movement range of severely affected stroke patients (Grimm et al., 2016), particularly when combined with NMES (Grimm and Gharabaghi, 2016).

However, the presented multifaceted device differs from previous approaches in several ways: the brain-controlled neurofeedback was not provided by an active robotic exoskeleton (Brauchle et al., 2015) but by NMES combined with a passive un-weighting exoskeleton (Meadmore et al., 2012; Hortal et al., 2015); in addition, NMES was not applied to proximal (Meadmore et al., 2012; Hortal et al., 2015) but to distal muscles (Meadmore et al., 2014), and was not controlled by kinematic information (Meadmore et al., 2012, 2014), but by physiological signals (Brauchle et al., 2015; Hortal et al., 2015) while applying a hybrid brain-machine interface (BMI) based on both sensorimotor cortical desynchronization (ERD) and electromyography (EMG) activity. Moreover, NMES induced no overt support (Meadmore et al., 2014; Hortal et al., 2015) but remained subthreshold (Grimm and Gharabaghi, 2016).

These modifications aimed to address limitations of current rehabilitation technologies, which usually take an all-or-nothing approach, e.g., by providing active robotic guidance to complete a movement as soon as the patient fails to reach the defined goal (Klamroth-Marganska et al., 2014); or by triggering NMES for overt muscle contraction, also referred to as functional electrical stimulation (FES), as soon as a predefined physiological state (recorded with either EMG or EEG) is achieved (Howlett et al., 2015). This all-or-nothing approach offers an important experience for patients who have not been able to move their hand or arm for years. From a motor learning perspective, however, it might be more successful to provide such rewarding feedback, e.g., robot-assisted movement of the paretic hand, only when a certain level of effort is made by the participant and gradually increased in the course of the training (Naros and Gharabaghi, 2015; Naros et al., 2016a). More targeted assistance might, therefore, be necessary during the rehabilitation exercises to maintain engagement without compromising the patients' motivation; i.e., by providing support—as little as possible and as much as necessary.

We, therefore, hypothesized that the adjustments implemented in our integrated approach provide assistance as needed to amplify the task-related ROM and the movement-related brain modulation during rehabilitation exercises of severely affected stroke patients without compromising their engagement.

## Methods

Patients were selected for this study when they were in the chronic phase after stroke (>6 months) presenting with a severe and persistent hemiparesis [modified upper extremity Fugl-Meyer-Assessment score (mUE-FMA) < 25]. Seven stroke patients (mean age: 59 ± 9.3 [41 89] years; 66.43 ± 16.6 [34 80] months post stroke; 14.3 ± 4.7 [9 23] mUE-FMA; male: female, 6:1; ischemic (middle cerebral artery): hemorrhagic stroke, 3:4; right: left hemisphere, 6:1). The mUE-FMA (without coordination, speed, and reflexes) was used to ensure that our results were comparable to earlier studies (Brauchle et al., 2015; Naros and Gharabaghi, 2015). This study, which was approved in accordance with the guidelines of the ethics committee of the local medical faculty, involved two sessions of wrist training with a multi-joint exoskeleton attached to the paretic arm. Each session consisted of approximately 30–40 movement trials with alternating wrist extension and flexion. Each movement period (extension or flexion) lasted for 5 s and was preceded by a 5 s rest period. This study is part of a larger research program on assisted reach-to-grasp movements in severely affected stroke patients. Within this framework, recent studies have revealed the importance of anti-gravity support with a multi-joint exoskeleton. We therefore applied this exoskeleton-based setup in this study as well to facilitate the transfer of the present findings into the overall research program. The exoskeleton and virtual reality have been described in detail elsewhere (Grimm and Gharabaghi, 2016; Grimm et al., 2016) and are cited here where applicable.

### Exoskeleton and virtual reality

We used a commercially available (Armeo Spring, Hocoma, Volketswil, Switzerland) rehabilitation exoskeleton for shoulder, elbow and wrist joints with seven axes (i.e., degrees of freedom) to provide antigravity support for the paretic arm and to register movement kinematics and grip force. Unweighing was realized via two springs that were incorporated into the device. This device could be used to make individual adjustments of, for example, the gravity compensation, thereby supporting patients with severe impairment in performing task-oriented practice within a motivating virtual environment. We extended these features in-house by using the real-time sensor data of the exoskeleton to display a three-dimensional multi-joint visualization of the user's arm in virtual reality. This entailed the use of a file mapping communication protocol to capture the angles of all arm joints and the grip force from a shared memory block. The virtual arm engine was programmed in a Microsoft XNA™ framework. The arm model utilized by the engine was constructed as a meshed bone-skin combination with 54 bones (3Ds Max 2010™, Autodesk). Using the measured joint angles and grip forces of the device, the bone-vectors of the meshed model were modified according to the movements of the user to provide online closed-loop feedback. The joint angles of the exoskeleton were directly represented in virtual reality, whereas the grip forces were augmented (i.e., amplified in virtual reality on the screen) to feedback natural hand function. This allowed visualizing finger movements on the screen, even though this information was not used for the study. However, the three-dimensional visualization of the fingers and wrist was applied during each task as an implicit online feedback of the movement. Prior to each session, participants were instructed to perform a natural wrist movement during the tasks aiming at maximum extension and flexion, respectively. The ROM of wrist movement was calculated as the sum of maximum extension and flexion and computed as the mean of each session.

### Neuromuscular electrical stimulation

**Figure 1:**
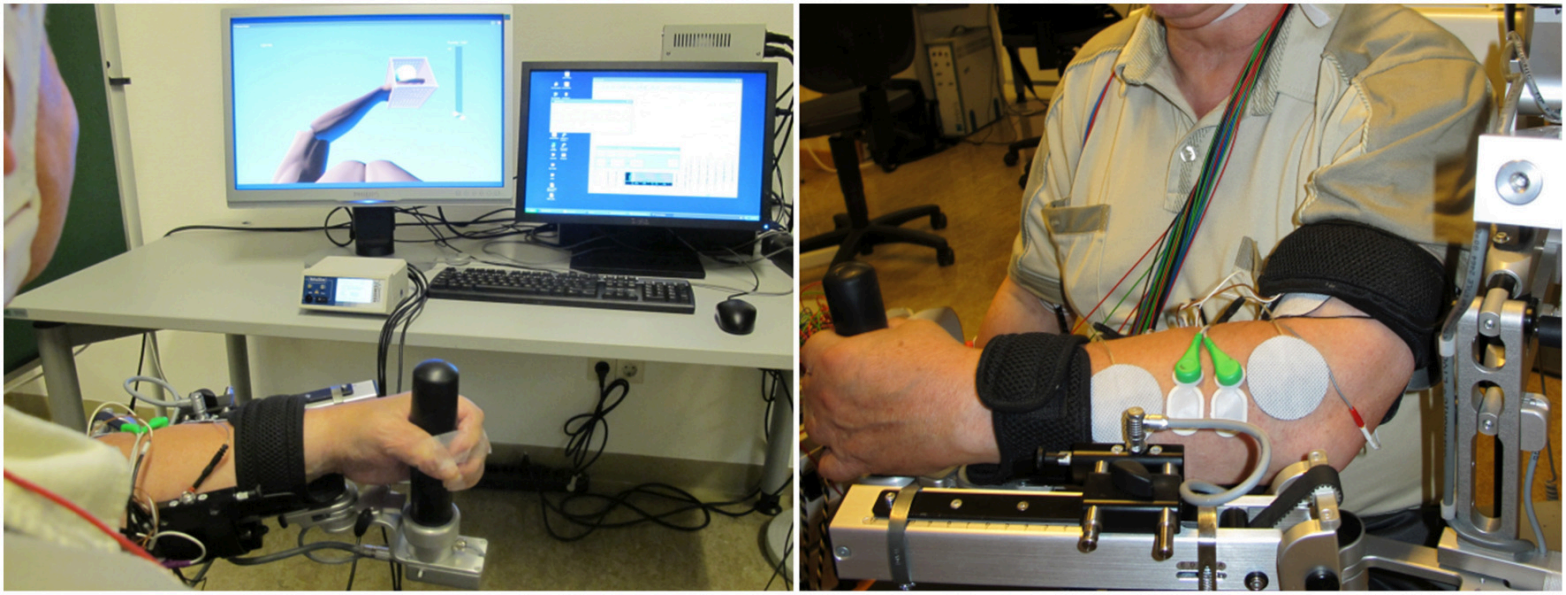
**Integrated neuroprosthesis with feedback via a virtual environment**. Assistance is provided by a gravity-compensating, seven degree-of-freedom exoskeleton attached to the paretic arm. Neuromuscular electrical stimulation is applied to the wrist extensor and flexor muscles during the exercises and is controlled by a hybrid brain-machine interface based on both sensorimotor cortical desynchronization and electromyography activity.

We integrated a NMES (De Marchis et al., 2016) device (Rehastim, 8- channel stimulator, Hasomed GmbH, Magdeburg, Germany) into the exoskeleton-based training environment with two bipolar, self-adhesive electrodes (diameter: 40 mm), and applied biphasic square impulses (frequency: 30 Hz, pulse width: 500 μs). The stimulation of this integrated neuroprosthesis (Figure 1) was updated in a closed-loop, real-time iteration at 60 Hz via a Controller Area Network/Universal Serial Bus (CAN/USB) port using a custom-made algorithm. Whenever the BMI classifier output was positive (see below), NMES was applied for 3 s to the M. extensor carpi ulnaris during wrist extension or to the M. flexor carpi radialis during flexion movement.

Each patient performed two exoskeleton-supported training sessions—one with and one without BMI-controlled NMES. Both the exoskeleton and the maximum stimulation intensity (Stim_max_) were individually calibrated. The exoskeleton was adjusted to provide optimized gravity compensation for every joint and to allow for unrestricted wrist movements in three-dimensional space. The Stim_max_ for each muscle group was empirically determined as the output current approaching the motor threshold but that was still perceived as comfortable. Since all participants suffered from severe upper limb impairment, prolonged supra-motor threshold stimulation was perceived as painful and was therefore not applied. The stimulation intensity was thus set in accordance with each patient's comfort level and just below motor-threshold, i.e., no visible joint movement, and resulted in a mean of 10.5 mA (±4.4 mA) and 9.5 mA (±4.4 mA) for the wrist flexor and extensor, respectively.

### Data acquisition

Electroencephalographic (EEG) signals were recorded with BrainAmp DC amplifiers and an antialiasing filter (BrainProducts, Munich, Germany) from 32 Ag/AgCl scalp electrodes (sampling rate: 1000 Hz) in accordance with the international 10–20 system (FP1, FP2, F3, Fz, F4, FC5, FC3, FC1, FCz, FC2, FC4, FC6, C5, C3, C1, Cz, C2, C4, C6, CP5, CP3, CP1, CPz, CP2, CP4, CP6, P3, POz, P4, POz, O1, O2; reference: FCz, ground: AFz). Electrode impedances were maintained below 10 kΩ. Since it often exceeds the frequency range of the physiological signals, ambient noise may compromise the recordings. To avoid an aliasing error due to undersampling of this noise, we, therefore, made every effort to remove all potential sources of electrical noise from the experimental environment, i.e., the high-frequency noise was deliberately avoided during the experiment and verified offline. Thanks to this approach, we observed no high-frequency noise in our recordings (Gharabaghi et al., 2014a; Vukelić et al., 2014; Bauer et al., 2015; Naros and Gharabaghi, 2015; Vukelić and Gharabaghi, 2015a,b).

Since EMG contaminations via compensatory movements are known to compromise EEG-based BMI training (Gharabaghi et al., 2014b), experienced examiners, who were trained to recognize these artifacts, instructed the patient to minimize them. As in previous studies with healthy subjects (Vukelić et al., 2014) and severely affected stroke patients (Naros and Gharabaghi, 2015), the patient was also instructed to avoid blinking, chewing, and any head and body movements other than the wrist movements. Together with visual inspection and feedback by the examiner, this approach proved to be a feasible method of preventing alternative BMI control. In addition, the EEG data was reanalyzed offline by visual inspection to remove all artifacted trials due to movement artifacts or current drifting; this resulted in a mean of 4.5 ± 3.8 excluded trials.

### Data analysis

Band pass (2–150 Hz) and notch filtering (50 Hz) were applied to the EEG raw signal. After epoching the filtered data into trials, visual artifact rejection was performed. This yielded an average of 26 ± 4 and 31 ± 3 (mean ± SD) trials in the non-NMES and NMES sessions, respectively. The power spectrum was normalized to the mean spectral distribution of the 5 s pre-movement rest period of the session. Mean movement-related spectral perturbation (ERSP) of the feedback electrodes were calculated for each session using the EEGLAB-Toolbox (Delorme and Makeig, 2004).

Surface electromyography (EMG) of the M. extensor carpi ulnaris and M. flexor carpi radialis were recorded with a band-pass filter of 0.1–1000 Hz and a sampling rate of 1000 Hz. The first task was used to set an individual EMG-threshold (area under the curve, AUC), to calibrate the EMG-classifier. Discrimination between movement and rest was performed by analyzing the activity of the measured EMG-channels. To this end, the EMG data of these channels was bipolarized and a Butterworth high-pass filter with an order of *n* = 2 and a cutoff at 1 Hz was applied. The waveform length $WL\left(t_{i}\right)=\sum_{t=t_{i}-w+1}^{t_{i}}\left|x\left(t+1\right)-x\left(t\right)\right|$ was calculated for each bipolarized EMG channel within a sliding window of *w* = 200 ms length. The sliding window was moved over the data in steps of 40 ms and corresponded to the waveform length of both channels. The waveform length feature of EMG has already been used to successfully decode different movements from EMG activity (Tenore et al., 2009). To correct for a delayed response of the subject to the cues, we calculated the cross-correlation of a vector *W* = *WL*(*t*_*i*) containing the waveform length feature with a vector P = P(t_i) which encodes the trial phase, where P(t_i) = 1 if t_i is part of the movement phase (otherwise 0). We used the latency of the maximum of the cross-correlation sequence as an offset to improve the assignment of the waveform length to the movement or rest class (*M*_*WL*_ or *R*_*WL*_, respectively). We identified the threshold T for the discrimination between the two distributions *M*_*WL*_ and *R*_*WL*_ with a Receiver Operating Characteristic (ROC) analysis. The criterion for threshold selection was set such that the false-positive rate was lower than 5% to ensure high specificity (≥0.95) of the classifier.

### Brain-machine interface (BMI)

**Figure 2:**
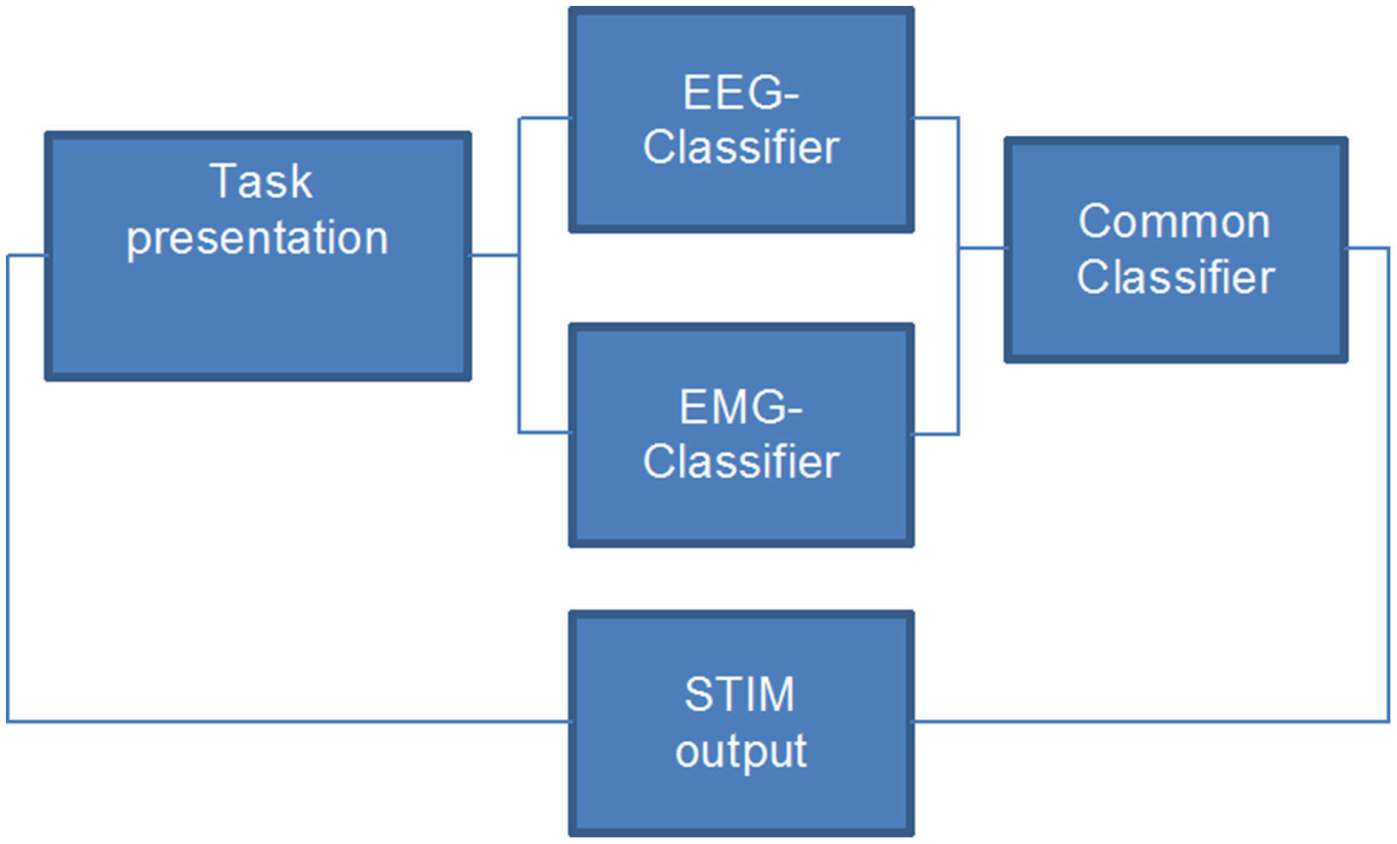
**Flow chart of the closed-loop hybrid brain-machine interface environment**. Neuromuscular electrical stimulation is applied only when both the EEG- and the EMG-classifier provide a positive output, i.e., when the task-specific effort of the participant is detected.

The BMI environment was designed to stimulate the patient's wrist during the movement (recorded by EMG) as soon as movement-related event-related desynchronization (ERD) in the β-band was detected in the ipsilesional hemisphere (Walter et al., 2012; Gharabaghi et al., 2014a). NMES stimulation was not triggered unless both the EMG and EEG classifier gave a positive output (Figure 2). We hypothesized that this hybrid approach improves the stability of classification (Leeb et al., 2011) and expected that the effects on ROM and ERD are bigger when using BMI+NMES than the exoskeleton alone.

During the NMES session, the same EMG filtering and feature extraction strategy as described above was employed. After bipolarization and filtering, the samples of each data packet from these channels were joined together to form a 200 ms-long queue. The waveform length was computed, summed up for both channels and compared to the threshold T for movement detection. If it exceeded T, the EMG classifier gave a positive output.

The EEG algorithm was based on the spectral power values between 16 and 22 Hz for three selected channels (FC4, C4, and CP4). We applied the same frequency-range and setup as in our previous BMI studies (Gharabaghi et al., 2014a; Vukelić et al., 2014; Bauer et al., 2015; Naros and Gharabaghi, 2015; Vukelić and Gharabaghi, 2015a,b). The spectral power was calculated using an autoregressive model order of 16 (McFarland and Wolpaw, 2008). This was fitted to the last 500 ms of the signal and updated every 40 ms. Classifier output was positive when 5 consecutive 40 ms epochs (i.e., 200 ms) were classified as ERD-positive. An epoch was not regarded as ERD-positive until the output of the classifier exceeded a threshold θ (Walter et al., 2012; Gharabaghi et al., 2014a; Naros and Gharabaghi, 2015; Naros et al., 2016a). The online signal processing was performed with the standard algorithm of the BCI2000 software (Mellinger et al., 2007). With a bin width of 2 Hz and targeted bin centers of 17, 19, and 21 Hz, the resulting frequency band was 16–22 Hz and corresponded to a wave length of between 45 and 62 ms. Choosing a data window of 500 ms enabled us to capture several cycles of these frequencies for reliable power analysis. This approach has already proved to be reliable in studies with the very same BMI setup (Walter et al., 2012; Gharabaghi et al., 2014a; Vukelić et al., 2014; Bauer et al., 2015; Naros and Gharabaghi, 2015; Vukelić and Gharabaghi, 2015a,b).

The sensitivity and specificity of the classifier of a linear discriminant analysis were indicated by the true-positive rate (TPR) and the true-negative rate (TNR), respectively; the false-positive rate (FPR) equaled 1-TNR. TPR and TNR were calculated by

(1) $$\begin{array}{l} TPR=\frac{\text{p}{\text{N}}_{\text{move}}}{{\text{N}}_{\text{move}}} \end{array}$$

(2) $$\begin{array}{l} TNR=\frac{\text{n}{\text{N}}_{\text{rest}}}{{\text{N}}_{\text{rest}}} \end{array}$$

with N as the total number of sample blocks in either the rest or move period, and pN and nN as the positively and negatively classified sample blocks, respectively.

The classification accuracy (CA) of a BMI system was defined by

(3) $$\begin{array}{l} CA=\frac{\text{TPR}+\text{TNR}}{2} \end{array}$$

and estimated for the different classifier modalities, i.e., EEG, EMG, and hybrid EEG/EMG. In addition, the correct response rate (CRR) was calculated as the ratio between the number of actions (i.e., BMI controlled NMES assistance) and the number of trials.

### Statistics

Statistical analysis was performed on a Matlab 2010b Engine. Data was tested for normal distribution using the Lilliefors-test (2-sided goodness-of-fit test). For normally distributed data, a dependent *t*-test for paired samples was performed; otherwise a Wilcoxon's signed ranks test was used. The significance level was set at *p* = 0.05 for all tests.

## Results

**Figure 3:**
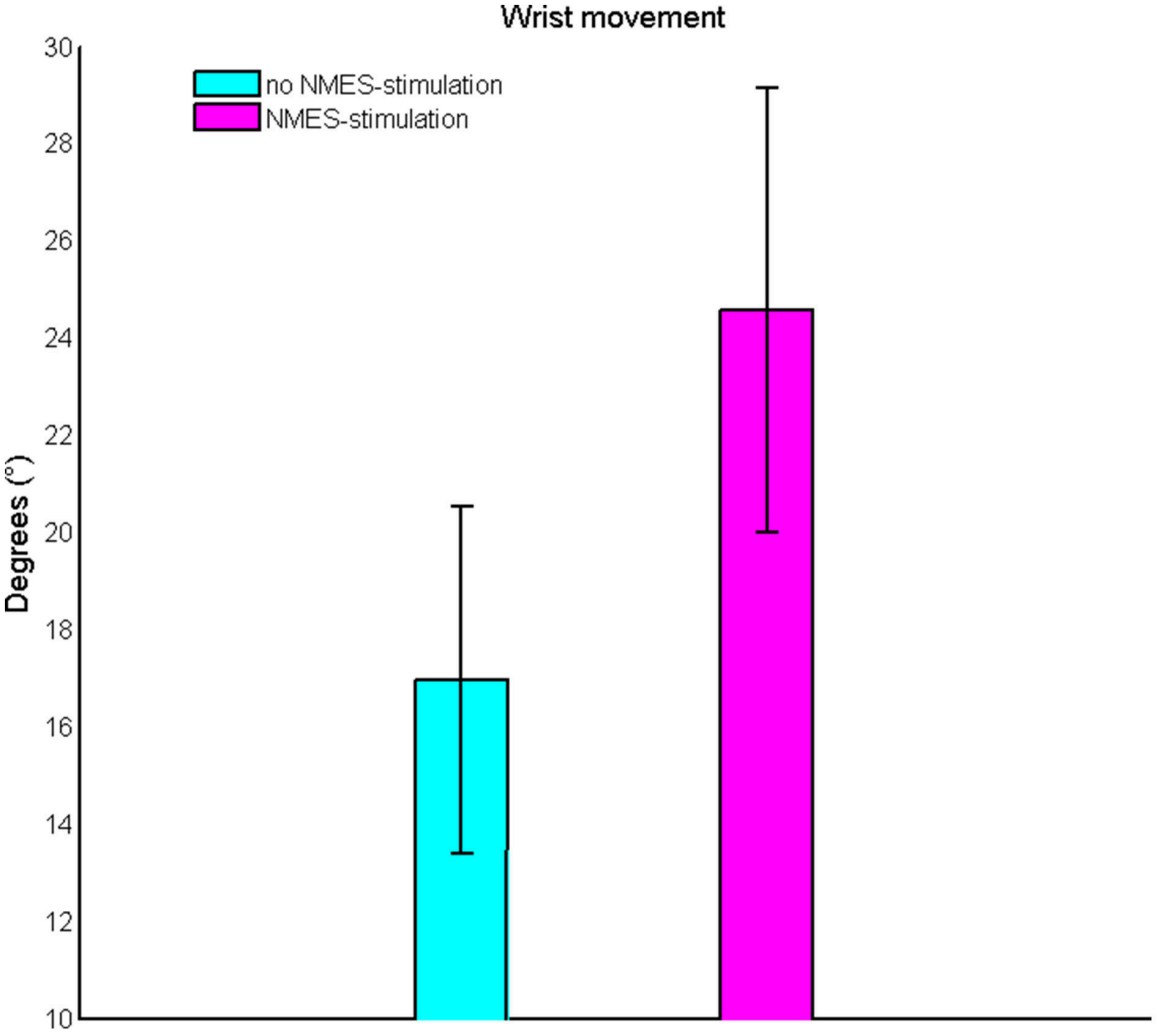
**Change of the task-related range of motion of the wrist**. Subthreshold neuromuscular electrical stimulation increases the range of motion on the group level.

Subthreshold NMES could be well integrated into the exoskeleton-based training; the effects on ROM and ERD were bigger when using BMI+NMES than the exoskeleton alone. More specifically, this combined approach increased the task-related ROM of the wrist from 18 ± 6° to 26 ± 8° (*p* = 0.009, Figure 3).

**Figure 4:**
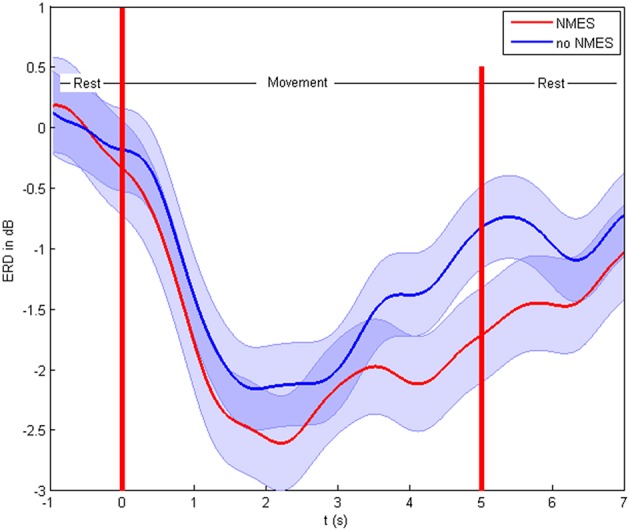
**Event-related desynchronization in dB**. Cortical activity and standard deviation in the feedback frequency band (16–22 Hz) as the average at CF4, C4 CP4 for the different conditions on the group level.

**Figure 5:**
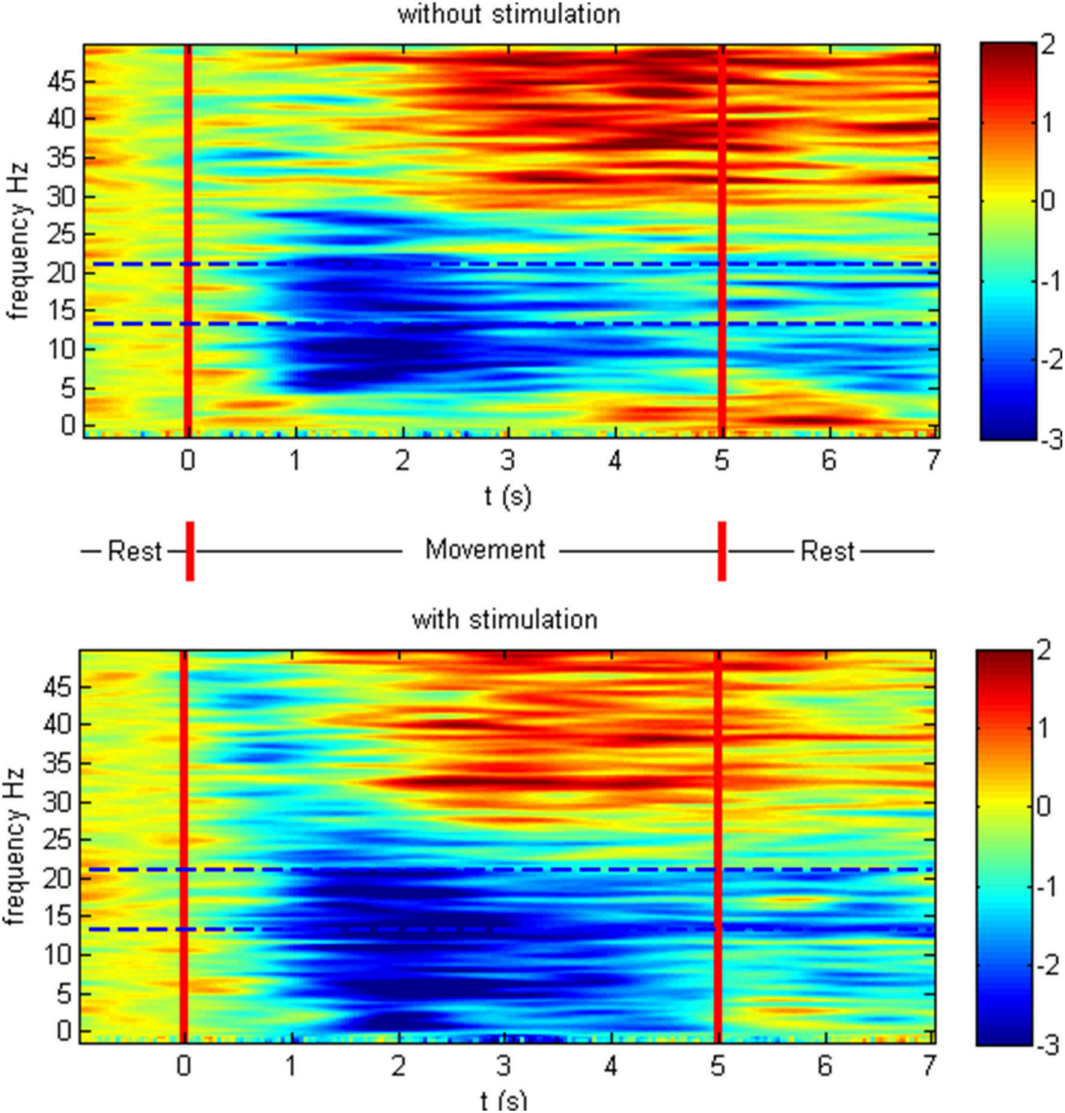
**Event-related spectral perturbation in dB**. Time-frequency plot of cortical activity as the average at CF4, C4 CP4 for the different conditions on the group level. The intervention modulated the movement-related brain activity by prolonged desynchronization in the feedback frequency band (16–22 Hz) indicated with dotted lines as well as by inducing additional broadband ERD throughout the task period in the low beta, delta, and gamma band.

The patients showed ERD both in the non-supported and the NMES-supported tasks. The ERD maximum for the decoded channels and frequencies was −2.47 and −2.83 dB in the non-supported and NMES-supported tasks, respectively. The intervention modulated the movement-related brain activity by amplifying the desynchronization (Figure 4) in the feedback frequency band (16–22 Hz) as well as by inducing significant (*p* = 0.019) additional broadband ERD throughout the task period in the low beta (14–16 Hz), delta (2–5 Hz), and gamma band (45–47 Hz) (Figure 5).

**Figure 6:**
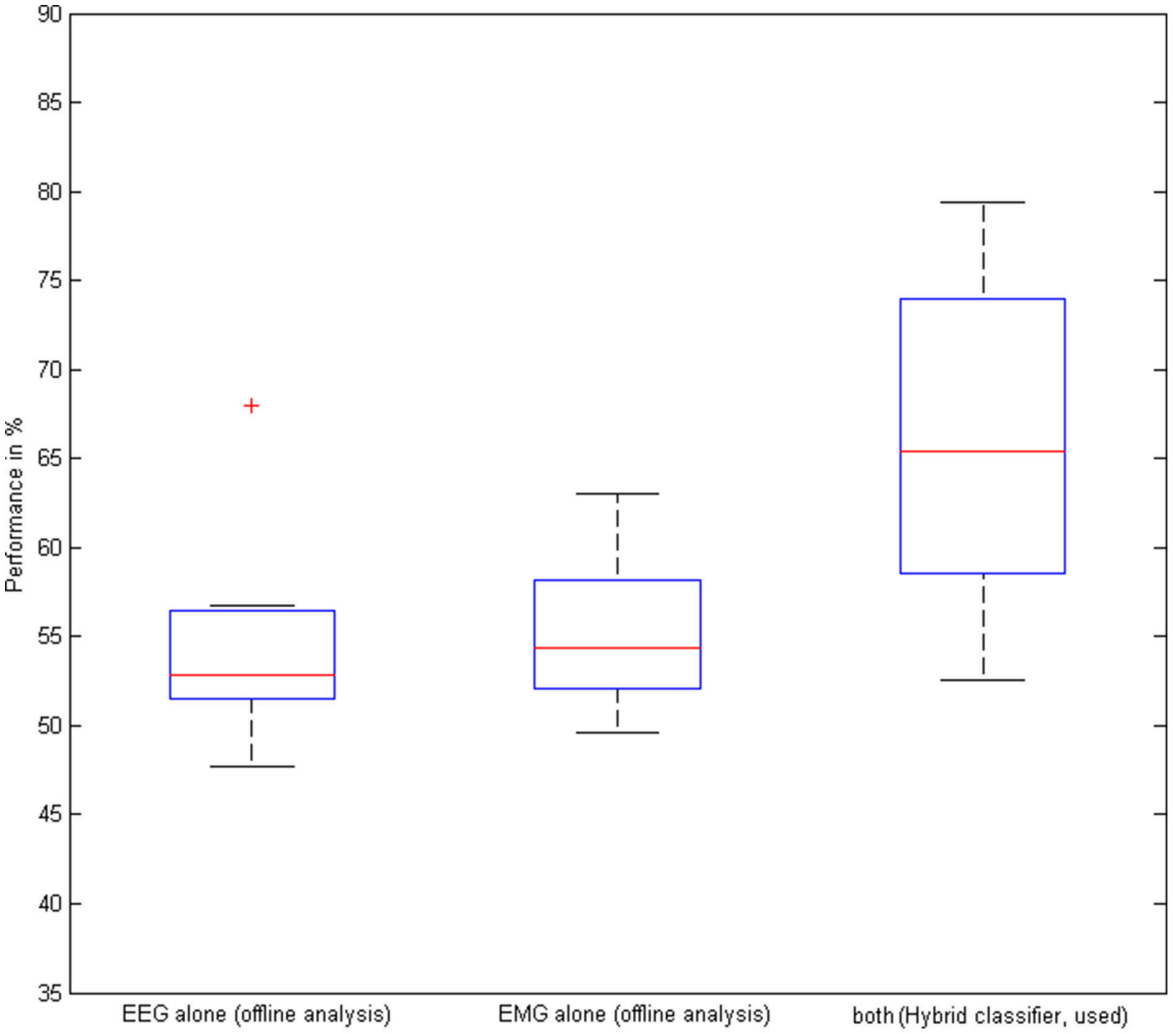
**Performance of the hybrid classifier**. Classification accuracy based on EEG, EMG, and EEG/EMG on the group level. The red cross indicates an outlier.

The hybrid BMI, i.e., combining the classification output of the EEG and the EMG classifier, was used during the task for online control. By achieving a mean classification accuracy of 66 ± 9.6% compared to 55 ± 6.4% (offline analysis with the EEG-classifier only) and 55 ± 4.6% (offline analysis with the EMG-classifier only, Figure 6), the hybrid BMI controlled the stimulation significantly better than either the EEG (*p* = 0.028) or the EMG (*p* = 0.021) modality. This gain was achieved by increasing the specificity of the classification, i.e., by significantly reducing the false positive rates to 22 ± 7.1% with the hybrid approach as compared to 37 ± 6.3% with the EMG (*p* = 0.037) and 53 ± 5.1% with the EEG modality (*p* = 0.007). On average, the device was triggered in 24 out of 31 trials, i.e., achieving a CRR of 77%.

This improved accuracy with the hybrid approach was also reached for the offline analysis of the non-NMES sessions (when no BMI or classification took place) by achieving 63 ± 6.9% compared to 56 ± 5.9% (EEG-classifier) and 55 ± 4.6% (EMG-classifier, Figure 6); the hybrid BMI classified significantly better than either the EEG (*p* = 0.031) or the EMG (*p* = 0.038) modality, notably, without a potential bias by the actual application of this classifier and the BMI-NMES during the task.

## Discussion

This proof-of-principle study has demonstrated the feasibility of an integrated neuroprosthesis combining a hybrid BMI—based on both cortical and muscle activity—with an exoskeleton and NMES for neurofeedback training via a virtual environment; this neuroprosthesis increased the ROM of wrist movement in chronic stroke patients with a severe impairment of the upper-extremity. Unlike other studies with similarly affected stroke patients, in which robots completed a movement initiated by the patients (Klamroth-Marganska et al., 2014; Brauchle et al., 2015), the technology applied here provided antigravity-support only (Housman et al., 2009), i.e., rendered no active assistance, thereby exploiting patient engagement and avoiding under-challenge during neurorehabilitation. However, future studies need to disentangle the contributions and mechanisms of BMI, NMES, and exoskeleton practice separately. Moreover, future intervention studies need to apply multiple sessions to explore whether cumulative increases of ROM and ERD can be achieved with this approach.

In this context, brain-controlled neurofeedback training aims to modulate cortical physiology and is applied to increase the responsiveness of the brain to subsequent physiotherapy (Pichiorri et al., 2015). When used in conjunction with commercially available robotic rehabilitation technology, these devices are also referred to as brain-robot interfaces (BRI; Bauer et al., 2015; Fels et al., 2015; Kraus et al., 2016a; Naros et al., 2016a). Such brain-robot interfaces can be applied for both restorative and assistive purposes. Even though both methods employ similar technology, restorative interfaces differ in concept substantially from brain-controlled assistive devices, which aim to compensate for lost function (Hochberg et al., 2012; Collinger et al., 2013). While the latter approach intends to maximize speed and classification accuracy for high-dimensional control (Spüler et al., 2014, 2016), the former aims to facilitate self-regulation of brain activity, which is considered beneficial for recovery and might ultimately lead to persistent functional gains (Naros and Gharabaghi, 2015). Such a restorative goal necessitates methodological specifications, e.g., in the areas of constrained feature space, regularized feature weights, cognitive load, feedback modality, and threshold adaptation to facilitate reinforcement learning of brain self-regulation and corticospinal connectivity (Bauer et al., 2016a,b; Bauer and Gharabaghi, under review). Proprioceptive feedback, for example, has been shown to enhance brain self-regulation of beta-band oscillations in comparison to visual feedback only (Vukelić and Gharabaghi, 2015a); these self-regulated beta-oscillations, in turn, correlated with the increase in corticospinal excitability following BRI training (Kraus et al., 2016a).

These specifications are, however, often not taken into consideration when brain signals are applied during rehabilitation practice, e.g., to control robotic devices or NMES. Instead, classification algorithms are applied to maximize accuracy in an unconstrained feature space, e.g., with support vector machines computing optimal features of an extended oscillatory frequency band, thereby resembling the approach usually chosen for assistive brain-interfaces (Hortal et al., 2015). Following the requirements of restorative neurofeedback training, e.g., providing feedback to beta-band ERD may, however, result in relatively low classification accuracy—as also observed in the present study—and frustrate the participants (Bauer and Gharabaghi, 2015a; Fels et al., 2015). This is particularly true of the severely affected patient group since movement-related beta-ERD in the ipsilesional primary cortex is compromised in stroke patients in comparison to healthy controls, i.e., the more severe the patient's motor impairment, the less beta-ERD (Rossiter et al., 2014).

In this context, we recently argued (Naros and Gharabaghi, 2015) that the fact that beta oscillations are less optimal for classification purposes—e.g., for differentiating movement-related brain states in many stroke patients—does not compromise but rather qualifies this physiological marker as a therapeutic target. We referred to an analogy to the concept of constraint-induced movement therapy in stroke patients, where the affected rather than the healthy body side is trained to facilitate restoration instead of compensation of motor function (Naros and Gharabaghi, 2015); and proposed that restorative neurofeedback training should follow the therapeutic goal of restoring the sensorimotor loop via improved beta-band modulation rather than aiming to train the brain state that enables the patient to control the exercising device best. The latter is a strategy that is implicitly followed when selecting individual frequency bands with best classification properties, i.e., that best separate the rest and the task condition (Hortal et al., 2015; Pichiorri et al., 2015).

Under these circumstances, complementary strategies such as continuous threshold adaptation (Bauer and Gharabaghi, 2015a; Naros and Gharabaghi, 2015; Bauer et al., 2016a) or hybrid classifiers that consider both brain signals and electromyography (EMG) activity (Leeb et al., 2011) are necessary to improve patient control over the training devices. The latter approach proved to be effective in the present feasibility study by increasing the classification accuracy from 55 to 66% with the hybrid BMI, compared to the EEG- or EMG-classifier, and resulting in 77% task-related neuroprosthetic support. Notably, this improvement was achieved by increasing the specificity of the feedback, i.e., by decreasing the false positive rate, which is particularly relevant for reinforcement learning with brain-interface based neurofeedback (Bauer and Gharabaghi, 2015a), since the considerable challenge of these exercises (Bauer and Gharabaghi, 2015b; Fels et al., 2015) might condition the patients to explore alternative, i.e., therapeutically undesirable, strategies (Gharabaghi et al., 2014b). Moreover, this hybrid approach enabled patients to achieve BMI controlled NMES assistance in more than 70% of the tasks, a level which is regarded as necessary for achieving a sense of self-efficacy during motor learning with assistive technology (Metzger et al., 2014). Notably, EMG signals alone were insufficient for classification in this study and might in general be inadequate as a control signal in the targeted patient group due to paralysis and/or abnormally co-activated muscles (Wright et al., 2014), a condition especially relevant in the severely impaired stroke patients who might benefit most from assistive rehabilitation technology.

Furthermore, the presented closed-loop framework facilitated the beta-band ERD, thereby adhering to the operant conditioning rationale, i.e., reinforcing the targeted activity considered to be beneficial for recovery and which might ultimately lead to functional gain (Bauer and Gharabaghi, 2015b; Naros and Gharabaghi, 2015; Naros et al., 2016b). However, whether this effect was achieved directly via the subthreshold NMES or mediated by the increased ROM in the NMES condition remains to be clarified. The spectral changes beyond the feedback frequency band suggest the former since the stronger wrist movement in the neuroprosthetic condition as compared to the orthotic condition is unlikely to result in broadband modulation of cortical activity in itself. Future studies, however, need to test this hypothesis by comparing different movement extensions with the same intervention, i.e., either neuroprosthetic or orthotic support. Importantly, recent findings indicated that NMES amplifies both ERD and cortical excitability when combined with motor imagery (Reynolds et al., 2015) or volitional effort (Stein et al., 2013). The facilitated ERD might, therefore, provide the substrate for future gains following repetitive application since the task-related ERD during brain-robot training have been shown to correlate with the cortico-spinal excitability after the intervention (Kraus et al., 2016a).

In recent approaches for stroke rehabilitation, patients controlled the rehabilitation robots with their brain signals, i.e., via motor imagery-related oscillations of the ipsilesional cortex, thereby successfully linking three-dimensional robotic training for reach-to-grasp movements to the participant's effort (Brauchle et al., 2015). The findings suggest, however, that sustained brain self-regulation for brain-controlled robotic training might be challenging (Brauchle et al., 2015) and may even be characterized by a significant association with the experience of frustration for the participants (Fels et al., 2015). To avoid this over-challenge, the brain-control assistance should probably be applied with more precision. In the same vein, complementary approaches applied NMES concurrently with antigravity support with a multi-joint exoskeleton (Meadmore et al., 2012; Hortal et al., 2015), thereby directly addressing the strength of specific muscle groups. However, these approaches stimulated proximal muscles of the upper limb, while the activation of wrist and hand muscle might be particularly important for functionally relevant improvements (Meadmore et al., 2014). The brain-controlled NMES in the present study has therefore been focused on wrist movement while continuous antigravity support via a passive multi-joint exoskeleton was provided to the rest of the upper limb.

In the context of neurorehabilitation, NMES is usually applied at supra-motor threshold intensity (referred to as FES) to train either arm or leg function; advanced approaches applied this stimulation to the upper extremity in conjunction with brain-interface technology for spinal cord injury patients (Pfurtscheller et al., 2003; Kreilinger et al., 2013; Rohm et al., 2013; Vučković et al., 2015) and stroke survivors (Ethier et al., 2015; Hortal et al., 2015).

In this context, the present study was the first to apply BMI-controlled subthreshold NMES to support the wrist exercises by extending the ROM in accordance with the actual ability of each patient. Importantly, to avoid under-challenge, stimulation was applied adjunct to voluntary contraction and not as an alternative. An additive stimulation approach such as this was shown to be effective for repetitive task practice of upper limb exercises in severely impaired, chronic stroke patients (Thrasher et al., 2008; Oujamaa et al., 2009; Mann et al., 2011). However, our neuromodulation paradigm remained subthreshold during the task, whereas the aforementioned NMES studies of the upper limb, even if physiologically triggered, followed an all-or-nothing concept with supra-threshold stimulation. Our state-dependent stimulation, which was controlled by the hybrid BMI, was, therefore, more subtle than in these earlier approaches. Due to the fact that functional muscle contraction was not realized by the stimulation itself, the increased performance was attained by modulations of self-initiated, orthosis-assisted movements. This outcome indicates an overall facilitation of sensorimotor networks by the subthreshold NMES and could constitute a novel restorative strategy in chronic stroke patients suffering from severe impairment of the upper extremity. Further research should investigate whether greater kinematic gains can be attained with other stimulation paradigms, such as the application of increased neuromuscular stimulation or concurrent transcranial current stimulation to facilitate exoskeleton-based motor leaning (Naros et al., 2016b). Our approach, however, led to kinematic gains while still encouraging our patients to participate. Progression of training is required to provide a further challenge for motor learning (Guadagnoli and Lee, 2004). This could be achieved either by means of a decrease in the NMES support level (Meadmore et al., 2014) or by automatic adaption of the level of training during robot-assisted stroke rehabilitation (Metzger et al., 2014). Both methods could in future be integrated into this neuroprosthetic set-up without difficulty and, by performing repetitive sessions within intervention studies, their respective clinical relevance in the targeted patient population should be examined more closely.

The neuroprosthesis introduced here holds the promise of bringing even more gains, e.g., via the simultaneous application of further interventions such as brain state-dependent cortical stimulation (Kraus et al., 2016b; Royter and Gharabaghi, 2016) to make full use of the salvaged restorative potential. Particularly, during exercises with severely impaired stroke patients, the task-related and muscle-specific facilitation that this device generates could provide the framework for concurrent cortical stimulation. For example, activity-dependent transcranial magnetic stimulation during robot-assisted training could provide such an additional input (Gharabaghi, 2015; Massie et al., 2015) Post-stroke latent corticospinal connectivity may be unmasked during brain-robot interface exercises by associative brain state-dependent stimulation (Gharabaghi et al., 2014a). As per Hebbian-like plasticity rules, such state-dependent stimulation synchronized to maximum gains of assisted ROM could consolidate the corticospinal circuits involved. More specifically, brain-robot feedback-based neuroprosthetic exercises may cause connectivity changes in cortico-cortical motor networks (Vukelić et al., 2014; Vukelić and Gharabaghi, 2015a) and result in a redistribution of cortico-spinal connections (Kraus et al., 2016a). Therefore, advanced assistive rehabilitation technology such as the one presented here could offer a backdoor to the motor system and provide better prospects of recovery (Bauer et al., 2015). When patients do not gain volitional control of this technology with beta-modulation via a standard EEG-based approach despite the strategies mentioned above (Naros and Gharabaghi, 2015)—e.g., due to an extended cortical lesion and distorted physiology—*epidural* recordings of field potentials may nonetheless facilitate the detection and neurofeedback training of this physiological target (Gharabaghi et al., 2014b). Such an approach closer to the neural signal source may also induce clinical gains after a shorter therapy time than is usually applied with the standard EEG technique (Gharabaghi et al., 2014c) and may even serve as a bi-directional interface for concurrent brain stimulation (Gharabaghi et al., 2014d).

In conclusion, during rehabilitation exercises, the combination of a BMI with neuromuscular stimulation and antigravity assistance has cumulative effects on both ROM and cortical modulation and, as such, may constitute a novel restorative framework for severely affected stroke patients while retaining their voluntary effort. Whether, such technological refinements also result in relevant functional gains will need to be investigated by comparing them in controlled intervention studies with dose-matched, conventional physiotherapy.

## Author contributions

FG participated in the study design and software development, supervised the measurement sessions and carried the data analysis. AW, MS, and WR participated in the software development. GN supervised the measurement sessions. AG participated in the study design and data analysis, and wrote the manuscript. Authors jointly drafted and approved the final manuscript.

### Conflict of interest statement

The authors declare that the research was conducted in the absence of any commercial or financial relationships that could be construed as a potential conflict of interest.
